# Lessons learned from the opioid crisis across the pillars of the Canadian drugs and substances strategy

**DOI:** 10.1186/s13011-019-0220-7

**Published:** 2019-08-19

**Authors:** Sheena Taha, Bridget Maloney-Hall, Jane Buxton

**Affiliations:** 1 Canadian Centre on Substance Use and Addiction, 500-75 Albert Street, Ottawa, ON K1P 5E7 Canada; 2 British Columbia Centre for Disease Control, 655 W 12th Avenue, Vancouver, British Columbia V5Z 4R4 Canada

**Keywords:** Lessons learned, Opioid crisis, Prevention, Treatment, Harm reduction, Enforcement

## Abstract

**Background:**

Canada is facing an urgent challenge to reduce the harms associated with opioids: from January 2016 to December of 2018, more than 11,500 individuals lost their lives due to opioid related harms. This review examines responses to the opioid crisis thus far, the lessons learned from these initiatives and the knowledge gaps that still need to be addressed across the four pillar model adopted by the CDSS.

**Methods:**

A search of peer-reviewed literature was conducted in PubMed and PsycNet, and grey literature was retrieved from reputable substance use and health organizations to determine responses to the opioid crisis and related outcomes between 2013 and 2019. Findings related to actions, outcomes and unintended consequences across the categories of prevention, treatment, harm reduction, enforcement and the evidence base were included and synthesized into a narrative review on lessons learned.

**Results:**

The opioid crisis is a result of multiple, complex interrelated factors. Many physicians may not feel competent to appropriately treat pain and/or addiction. Pushes for opioid deprescribing have resulted in some individuals using illicit opioids as treatment. A range of effective and accessible pharmacological and psychological treatments are still required. When regulations are barriers, unsanctioned actions, such as overdose prevention sites, may be enacted by individuals to respond to urgent public health needs. A nimble response with evolving enforcement perspectives can aid individuals experiencing harms from opioid use.

**Conclusions:**

There is no one size fits all response to this crisis, and consideration should be given to the unique needs of different communities and populations, as well as the broader impact of harms on families, communities, and society. A situation so multifaceted requires both immediate and long-term strategies implemented concurrently in order to address the differing and on-going needs of Canadians experiencing opioid harms. The expertise of individuals and families affected by the opioid crisis must be included in consultations and decisions related to different strategies, to ensure responses are not stigmatizing, that they will be effective and acceptable and that unintended consequences are quickly recognized and mitigated.

## Introduction

Canada is facing an urgent challenge to reduce the harms associated with opioids. From January 2016 to December of 2018, more than 11,500 individuals lost their lives due to opioid related harms [1], confirming that we are experiencing a national public health emergency [2]. These deaths represent a significant number of families, friends and communities that are grieving and working to prevent further devastation. Considerable efforts have been made across the country to address the crisis and minimize ongoing harms, yet it continues. This summary examines the responses to the opioid crisis thus far, the lessons learned from these initiatives and the knowledge gaps that still need to be addressed.

Learnings are organized along the Canadian drugs and substances strategy (CDSS), which adopts the four pillar model comprising: prevention, treatment, harm reduction and enforcement, all of which are grounded by a strong evidence base [3]. The prevention pillar of the CDSS focuses on preventing problematic substance use through increasing awareness of the dangers of substance use and decreasing the demand for substances [3]. Factors that globally prevent substance use harms include assessing and responding to the social determinants of health such as socioeconomic status, homelessness, familial attachment, education, and resiliency [4–6]. These approaches should include sex, gender, trauma and cultural considerations to ensure the well-being of all Canadians [7]. Treatment represents the range of options that should be available to support an individual if and when they choose to reduce or stop their opioid use [8–10], including pharmacological interventions such as opioid agonist therapy (OAT), as well as psychosocial interventions [11]. Harm reduction lessens some of the risks that can be experienced while using substances [3] and is about meeting people at whatever stage of the care continuum they may be at, and providing tools and resources to enable a person, their family and communities to be safer [12]. Harm reduction services also connect individuals to other supportive or treatment services to ensure their well-being and health [12]. Enforcement represents efforts responding to illegal drug manufacture and distribution [3].

Using this model we review responses taking place within each of the four pillars, although it is widely recognized that the complexity of the opioid crisis requires that action is also taken across the pillars. The Government of Canada acknowledges that the opioid crisis requires “a response that is comprehensive, collaborative, compassionate and evidence-based” [2]. This paper provides a timely summary of the actions and outcomes across the CDSS pillars.

It is important to reflect on the actions that have taken place so that we may consolidate the wealth of knowledge that has been gained up to now, recognize the strategies that are effective and in what context, acknowledge and routinely look for unintended consequences and identify the actions that still need to occur for an impactful response. Opportunities to share information and lessons learned are critical to ensure that Canada and other countries can develop and implement informed changes to drug policy, programs and practice to address the opioid crisis and harms from all substances.

## Methods

Given that the opioid crisis is still evolving, and that the literature is continuing to develop, this narrative review was intended to be broad and inclusive in scope [13]. It was determined that a literature review of peer-reviewed and grey literature would best capture emerging responses to the crisis.

### Search strategy

A literature search was performed by an Information Specialist using PubMed and PsycNET. Variations of search terms related to the opioid crisis, opioid epidemic, and Canada were used. The search was limited to English-language peer-reviewed articles published between 2013 and 2018 to ensure literature were relevant to the timing when the opioid crisis emerged and continued to evolve. Articles were restricted to those that addressed the Canadian context, though the studies may have included experiences from other countries as well.

Following this initial search, references of obtained papers were reviewed and scans of peer-reviewed literature published as of June 2019 were conducted to ensure the most recent responses to the opioid crisis were obtained. The search categories for this additional search comprised actions, outcomes, and unintended consequences across the pillars of prevention, treatment, harm reduction and enforcement, as well as the evidence base supporting these initiatives.

Grey literature that met the search categories outlined above and that was released between 2013 and 2019 was also retrieved from known reputable substance use and health organizations (e.g., Federal and Provincial government, World Health Organization). Grey literature was deemed reputable based on the publishing organization’s history of producing evidence-based reports, the clarity of stated aims and/or methodology, the relevance, and currency of the report [14–16].

### Study selection

The Information Specialist screened the results of the initial search and removed duplicates or any articles that were clearly outside of the scope of the project based on titles and abstracts. Of the 99 results, 65 were retained. The Research & Policy Analyst screened the 65 retained articles, the articles retrieved in the additional scan, and the grey literature to ensure they met at least one of the following inclusion criteria: a) addressed one or more of the pillars of the CDSS, b) outlined interventions implemented to respond to the opioid crisis, and/or c) discussed outcomes from a given intervention/policy change. Papers were excluded if they were irrelevant, or if they were written in language other than English. While the focus was on examining recent responses in Canada, international sources were included as other countries experienced opioid harms and have learnings that can be considered in the Canadian context. Sources older than 2013 were included to provide pertinent background information where newer publications did not exist. A formal quality assessment of each paper was out of scope for this project [13].

### Data extraction and synthesis

Data was extracted by identifying: a) the pillar of the CDSS the findings corresponded to, b) the population that was involved (e.g., physicians, individuals using opioids, individuals receiving treatment for opioid use disorder, etc.), c) the intervention or policy implemented, d) outcomes, and e) unanticipated consequences. These findings were synthesized into lessons learned categoriezed along the pillars of the CDSS.

## Results

### Prevention

Preventing harms from opioids can be considered from two streams. The first focuses on effective and appropriate pain management for individuals living in chronic pain [17]. The second focuses on preventing harmful use among those who use illicit opioids or prescription opioids for non-medical reasons [18].

#### Management of chronic non-cancer pain

Rates of opioid prescribing for chronic non-cancer pain began increasing in North America in the 1990s. Indeed, the volume of opioids sold to Canadian hospitals and pharmacies has increased by more than 3000% between the 1980s and 2000s [19]. More recently, the dispensing rate for high-dose formulations of several opioids, including morphine, hydromorphone, oxycodone, and fentanyl, increased by 23% from 2006 to 2011 [20]. This rise has been attributed in part to pharmaceutical companies misrepresenting the addictive potential of opioid medications to prescribers, dispensers, and patients [21, 22]. There is evidence that high levels of pharmaceutical marketing of prescription opioids is associated with higher levels of opioid prescriptions and opioid-related mortality in the United States [23] and the increasing rates of opioid prescribing has been linked to increasing levels of nonmedical opioid use [24].

Actions have been taken to respond to these issues, with guidelines, tools and templates developed to provide parameters around opioid prescribing and deprescribing, and to include the current best evidence available in this regard. For example, a recent meta-analyses suggest that alternatives, such as nonsteroidal anti-inflammatory drugs may have similar efficacy to opioids in achieving pain relief and improving physical functioning over the short-term [25]. Indeed, the first recommendation of the 2017 Canadian Guideline for Opioids for Chronic Non-Cancer Pain was to consider non-opioid and non-pharmacological treatments for individuals with chronic non-cancer pain before starting a trial of opioids [17].

Two inter-related lessons were learned from these findings: a) that a great number of individuals are living with chronic pain, with estimates ranging from 15 to 29% of the Canadian population [26] and b) that many physicians did not know how to respond to patient pain complaints, which may be a result of inadequate training [27]. In fact, a 2009 study found that while veterinary training programs had an average 87 h of mandatory pain content time, medicine programs had an average of only 16 h, and pharmacy programs had a mandatory 13 h [28].

Improved curriculum and continuing medical education on pain management and substance use disorders are needed to ensure the competency of prescribers and dispensers [27, 29]. Indeed, some research has shown that physician education can significantly decrease the number of opioid prescribed post-surgery [30]. Part of this education can also include raising awareness among health care providers on how their own stigma may affect treatment of people who use drugs [31, 32].

While the impetus to save lives is a motivator to take swift action, another lesson learned is that taking actions too quickly without considering all possible consequences can increase or create new harms. Prescription monitoring programs have been identified as one component to address the opioid crisis [33], yet there is limited evidence on their effectiveness in reducing harms [34]. Studies have revealed that some physicians reduced their rates of opioid prescribing due in part to fear or punitive action from their regulatory colleges [35–37]. Indeed, the defined daily doses of opioids prescribed have decreased across most of Canada between 2012 and 2016 [20]. However, this action led some individuals who were taking opioids to manage their pain to access the illicit supply when their prescription was suddenly cut off [38]. Deprescribing increased the dangers to individuals taking substances that were not pharmaceutical grade, of unknown content and potency, and which could contain dangerous contaminants such as fentanyl and its analogues. Deprescribing also caused some individuals to perform an illegal act to receive the pain relief previously provided by a physician prescribed medication. Furthermore, these actions culminated in individuals living with chronic pain to feel stigmatized for their initial pain condition, and then again for using illicit substances to manage their disorder [39, 40].

#### Non-medical use of opioids

Increases in prescribing not only affected those who were dispensed medications, but also increased the prevalence of prescription opioids in the illicit market due to diversion and theft of these medications [41]. Recent estimates indicate that 9.6% of Canadian adults who used opioid medications in 2018, reported some form of problematic use (e.g., taking in amounts greater than prescribed, tampering with the product before taking it, or using to get high or improve mood) [42]. As with those individuals who sought illicit opioids for chronic pain relief, individuals who used prescription opioids non-medically also had to increase their use of an illicit non-prescription grade supply when prescribing and diversion decreased [43].

A recent study found that non-prescribed opioids, including fentanyl, were playing a growing role in opioid poisonings, particularly in British Columbia. In the 2015–2016 fiscal year, only 34.1% of all opioid-related hospitalizations in British Columbia were among people with an active opioid prescription, a decrease from the 44.4% in the 2013–2014 fiscal year [44]. Indeed, in 2018, it was estimated that fentanyl was present in 85% of illicit drug overdose deaths [45].

Thus, a lesson learned is that preventing harms through changing prescribing practices is not sufficient to address the current crisis, and in fact, in some instances, had unintended negative consequences. Effective responses to reduce opioid harms, regardless of how opioid use was initiated, will require a comprehensive prevention strategy that addresses the physical, mental and social needs of an individual [46, 47].

### Treatment

Access to care is determined by affordability, availability, acceptability, accommodation and accessibility [48]. Barrier to treatment include wait lists [11] and accessibility of treatment supports particularly in areas outside of urban settings, and most significantly for some Indigenous populations in remote or fly-in communities [49]. As with prevention, it is also imperative that treatment services are culturally appropriate, adequately address the social determinants of health, and provide treatment for an individual’s mental and physical needs in an integrated manner [10].

A lesson learned is that various measures need to be taken to increase access and to make treatment services more connected during the opioid crisis. Emergency treatment funding committed investments from the federal Government, provinces and territories so that they could tailor the evidence-based treatment services to the needs of their populations or increase capacity to prepare for future impacts, with interventions like youth hubs, telemedicine, and on-the-land healing camps being funded [50]. Rapid Action Addiction Medicine (RAAM) clinics, assertive community treatment and other outreach efforts have been also utilized in Canadian jurisdictions to provide increased access to addiction treatment [10]. The use of these programs in North America have contributed to reduced emergency department visits, reduced wait times and lessened stigma [51], and greater engagement in treatment [52]. However, long-term evaluations of these interventions are still required.

Another lessons learned is that greater capacity was required in the health care system to provide comprehensive treatment services [53] - an issue that has been addressed in part by increasing the capacity of primary care providers and establishing connections between services. In 2018, the Canadian Research Initiative in Substance Misuse developed *National Guidelines for the Clinical Management of Opioid Use Disorder* [54]. These guidelines recommend buprenorphine as the first line pharmacological treatment for most individuals – a prescription and induction that can be performed by primary care physicians or nurse practitioners [54]. Additionally, the federal government removed the section 56 exemption required to prescribe methadone, allowing any medical professional to utilize this treatment option and thereby removing the onus on the patient to access a specialized clinic [55]. However, a lesson learned is that regulatory changes are not enough. Though permissions have changed, jurisdictional professional colleges may still restrict methadone prescribing [54], and permission changes do not address physicians lack of competence to manage individuals living with an opioid use disorder [56]. Moreover, even with increased access to these primary care-based treatment options, retention remains a challenge. A recent study in Vancouver found that only a third of study participants were retained on OAT in 2016 [57]. This suggests that current OAT options may not meet the needs of a majority of individuals who initiate treatment.

A recovery-oriented system of care may reduce barriers to individuals accessing and remaining in treatment [58]. Individuals who are living in recovery have taught that multiple services, both professional and informal, provide an individual with recovery capital: the critical supports that help individuals achieve their desired outcomes [59]. These same participants cite a lack of mental health and culturally appropriate services as well as the cost of all services, as barriers to recovery [59]. Individual, family or group psychosocial interventions can be effectively provided alongside pharmacological treatment but more research is needed on the efficacy of various therapies and if certain modalities correspond better to particular medical-treatments [10]. Of course, even with better knowledge of what works, individuals still need to have access and availability of these quality services.

### Harm reduction

Opioid-related harm reduction efforts in Canada have included safer consumption sites (SCS), overdose preventions sites, drug checking services, and overdose reversal kits (naloxone), to name a few [60]. These services can reduce the risk of disease transmission and overdose deaths so that Canadians who use drugs can be healthier and can continue to contribute their communities.

The evidence related to the effectiveness of SCS to prevent overdose, provide access to sterile needles and other drug use equipment, and connect individuals to support services and treatment has been established for some time [61, 62]. However, stigmatizing attitudes about drug use and harm reduction remain [5, 63]. In many cases, stigma is perpetuated by common language used to discuss substance use that is driven by moral opinion rather than by evidence [64]. The moralistic messaging associated with terms, such as “addict”, “drug abuse”, and “dirty” have contributed to the delayed widespread implementation of evidence-based harm reduction strategies, such as SCS, by implying that substance use is a choice and a personal moral failing, rather than a public health issue [63]. Furthermore, while members of the public may see the benefits of SCS for those who use drugs, they may still be reluctant to support a site in their neighbourhood [65].

While stigma is informed by many societal factors, it has been reinforced by the continued criminalization of drugs and drug use [66–68]. Moreover, stigma is impacted by an individual’s understanding of the causes of substance use disorders (i.e., degree to which it is a personal choice) and the perceived level of control an individual has in changing their substance use patterns [69]. Therefore, one component to combat stigma is by providing education about social and biological influences as precipitating factors to substance use disorders [70].

Another lesson learned is that when regulations cause delays in implementing responses necessary to reduce acute harms, individuals in the communities affected may take immediate action. In the wake of the opioid crisis, numerous unsanctioned supervised consumption sites were opened in cities across the country by volunteers. These “pop-up” sites, referred to as overdose prevention sites (OPS), addressed an unmet need as groups worked to receive exemptions from Health Canada to establish a sanctioned SCS [71], and indeed have averted opioid-related deaths [72]. In December of 2017, the Federal government recognized the urgent public health need and provided temporary class exemptions for OPS to be set up by volunteers in the provinces and territories [73]. Yet, where peers may volunteer to fill gaps in services, much of this work in under resourced and unsupported, resulting in a great emotional toll on these individuals [74, 75].

We have also learned that the wide-spread availability of naloxone without a prescription across Canada [76], with free take-home programs in all jurisdictions [77], has certainly saved lives. A recent study estimated that one death was averted for every 11 take-home naloxone kits used in British Columbia [72]. In British Columbia, Alberta, and Manitoba take-home naloxone kits distributed to community members have been used to reverse approximately 12,000 opioid poisonings [19]. When examining actions in BC alone, since 2012, more than 30,000 take-home Naloxone kits have been reported as used to reverse an overdose [78].

Drug checking services, wherein individuals can determine if there are contaminants, such as fentanyl, in the drugs they are planning to consume, have been recommended as one avenue to prevent poisonings [79, 80]. Various technologies are used for drug checking, including lower-cost options (e.g., fentanyl urine test strips) and more advanced laboratory techniques (e.g., mass spectrometry) [81]. There is limited evidence of the impact of drug checking services on substance use behaviours [81–84]. It is important that individuals are aware of the limitations of drug checking technologies, including that fentanyl test strips may not detect all fentanyl analogues, including carfentanil [85]. At the very least, it is clear that drug checking services create an opportunity for communication and education between harm reduction works and individuals who use drugs [81, 83]. The data collected from drug checking services provides an important window into the types of drugs and drug combinations being used in a given community, which could be a useful component of a substance use surveillance system [81, 83]. This detailed information could lead approaches that are tailored to the needs of communities and their residents.

### Enforcement

The single biggest lesson learned in the enforcement pillar is that arresting individuals who are using drugs will not end the crisis [86, 87]. While enforcement efforts focusing on production and distribution of illicit substances and unlawful distribution of controlled substances is a component of the CDSS [3], many policing bodies have recognized that arrest and incarceration are not the appropriate routes to prevent or address drug use on an individual level [87].

Some enforcement communities have undergone a paradigm shift, increasingly acknowledging harmful substance use as a chronic health issue rather than a criminal justice one. Many officers now see their role to be connecting individuals experiencing harms from opioid use to services, as opposed to enforcing correctional repercussions [88]. To support this notion, the federal Good Samaritan Drug Overdose Act became law in 2017 [89]. This Act allows an individual who has overdosed to receive emergency medical care while ensuring some legal protection related to simple possession of a controlled substance for personal use. This protection also applies to the individual(s) who has not overdosed but call emergency services [90].

The opioid crisis and resulting harms highlight the need for enforcement and regulations to be nimble, as substances of use are constantly evolving. Previously, when the Canadian Border Services Agency suspected parcels were being used to import drugs, they were only permitted to inspect packages that were large in size. As the potency of fentanyl allows small volumes in transit to be of concern, Bill C-37 amended the *Customs Act* to allow border security agents to inspect packages less than 30 g [91]. This bill also prohibits the unregistered import of pill presses and encapsulators, and allows new psychoactive substances to be scheduled and controlled quickly, to respond to the emerging nature of novel psychoactive substances.

### Evidence base

An effective response to the crisis requires comprehensive and robust monitoring systems to be able to measure emerging trends in substance use, harms and outcomes [92]. Without a complete understanding of where multiple factors stand as a baseline, it is impossible to know the gravity of the current situation or to track effects. A lesson learned is that developing useful monitoring systems requires collaboration to share data across Canada. There have been some positive examples of increased collaboration and data sharing to respond to the opioid crisis. For example, initially the comparability of opioid-related coroner’s data across the provinces and territories had been limited [93]. Improvements in recent years have increasingly allowed for opioid-related deaths to be categorized in the same way across jurisdictions, allowing for accurate national counts [94]. In addition, Health Canada’s Drug Analysis Service (DAS), which analyzes the contents and quantities of drug samples submitted by law enforcement across Canada [95], has recently begun sharing their analyses with the jurisdictions on a monthly basis, which contributes to a jurisdiction’s ability to detect emerging drug use trends. As a final example, a national drug checking working group was established in 2015 as a means to share emerging best practices and lessons learned among Canadian drug checking service providers [96]. Combined, the data from each of these sources exceeds their individual utility as together they disentangle the many complex factors related to opioid harms.

A lesson learned is that several avenues have to be taken to improve access to and the quality of data that can inform responses. In 2016, British Columbia’s provincial health officer declared a public health emergency in response to opioid-related deaths [97]. This declaration allows for data to be collected across the health system, and analyzed immediately to inform where action needs to be taken. British Columbia, Alberta and other jurisdictions have formed multidisciplinary groups to ensure the sharing of information across sectors and coordinated actions that are supported by increased investments [97–99].

## Discussion

As evident throughout this paper, there are gaps in our understanding and the application of effective strategies across the four pillars. Evaluations of the strategies that are currently being implemented to respond to the crisis are critical to ensuring that future actions are evidence-informed. For example, educational efforts to improve practitioner competence need to be evaluated to ensure that physicians, nurse practitioners, dentists, pharmacists and all related health care providers are competent to respond to the pain and substance use needs any client may have. New models of treatment provision, such as RAAM clinics, require outcome evaluations to ensure they are beneficial for all involved.

Going forward, research is required to establish a better understanding of chronic pain, which pain management strategies are most effective for which individuals and under what circumstances, and who may be most at risk of developing an opioid use disorder, so that services and supports can be put in place that are evidence-based [26, 100]. In short, we are still learning how to respond to the need for a broader range of treatment options that are better able to engage those in need of supports. Additionally, more research is required to refine the evidence base on harm reduction principles, and address additional controversial topics such as inhalation and assisted injection methods of administration, access to an uncontaminated supply of drugs such as diacetylmorphine and hydromorphone, and decriminalization of psychoactive substances.

Even when data are available, proposed responses must be examined for unintended consequences and informed by the expertise of people with lived and living experience [74]. For example, embedding alternative pain management strategies such as physical therapy and cognitive behavioural therapy into healthcare services have been promoted as one component of the response and have become more wide-spread [101–103]. Yet, these services often require multiple treatments to obtain a benefit, and remain out of reach for many Canadians, as they are not all covered by all provincial health care plans or may not be available in all regions of Canada. Additionally, findings from coroner assessments has revealed that many opioid-related deaths are occurring among individuals who are using opioids alone in their homes [104, 105], resulting in public health messaging for individuals to ‘not use alone’ [106].This recommendation may not meet the needs of individuals who use drugs as they may prefer to use their substances alone in the comfort of their home or, the restrictions at consumptions sites may not allow them to use their preferred method of administration (e.g., inhalation) [107]. As well, drug checking services must be tailored to a community’s unique needs and implementation needs to be cautious of unanticipated outcomes. In communities where fentanyl is occasionally found as a contaminant, fentanyl test strips can be an invaluable drug checking tool, whereas in communities where fentanyl is present in a majority of the drug supply, these test strips are less useful. Moreover, test strips that are not able to detect carfentanil or new analogues, may have the unintended consequence of providing individuals with a false sense of security regarding the substance they plan to consume. These examples highlight the importance of involving individuals who use(d) substances in all conversations about solutions to ensure their needs are truly met and that proposed options are acceptable, accessible, available, accommodating and affordable.

In recognition of this, the evidence for this review came from academic literature, experiential evidence and the expertise of individuals with lived and living experience represented in the grey literature. This review included and equally weighted peer-reviewed literature and grey-literature to a) value to voices and experiences of all individuals responding to the opioid crisis, even those who may not be connected to academic publishing, and b) reflect responses occurring at the grass roots level that may not be represented in peer-reviewed literature.

### Limitations

Our understanding of the opioid crisis and its precipitating factors have continued to unfold over time revealing a complex multi-disciplinary problem [60]. A limitation of this paper is that the long-term effects of the strategies and programs outlined within are not yet known. As more evidence in generated on responses to the opioid crisis, a systematic review would be warranted. Despite efforts to comprehensively search the literature, it is possible that relevant records were not included in this analysis due to language or database restrictions. Articles were retrieved from two databases that are known to be relevant to the field. It is possible that by restricting the search to these databases, articles may have been omitted that could have address other perspectives on the opioid crisis (e.g., sociological, legal, etc.). An additional limitation is that a quality assessment of the articles included was out of scope for this narrative review [13].

As the literature related to the opioid crisis is continuing to evolve, and because many response are taking place at the community level, grey literature was included to ensure that this review contained the most recent and relevant reports. While not part of a systematic search, excluding this data would have provided an incomplete and inaccurate picture of the current crisis. A limitation of this approach is that the grey literature included in this synthesis may not have been peer-reviewed. To mitigate the risk of low-quality findings, grey literature was only retrieved from reputable sources (e.g., Canadian Institutes of Health Research, World Health Organization). Given the grassroots responses to opioid harms, there may be additional lessons learned that are not represented in the peer-reviewed or grey literature, which limits the conclusions drawn from this review.

## Conclusion

This summary has outlined lessons learned within each pillar of the CDSS, but it is important to note that actions taken based on these learnings should not be discrete. Collaboration across sectors and all levels of government is required to ensure responses are complementary and not siloed. Yet, there is no one size fits all response to this crisis; consideration needs to be given to the unique needs of different communities, Indigenous peoples, youth and correctional populations, sex and gender issues, as well as the broader impact of harms on families and society.

A situation so multifaceted requires both immediate (e.g., widespread availability of naloxone) and long-term strategies (e.g., addressing social determinants of health) implemented concurrently in order to address the differing and on-going needs of Canadians. The ultimate lessons learned is that the expertise of individuals and families affected by opioid use must be valued and incorporated into decision-making to ensure responses are not stigmatizing, that they will be effective and acceptable, and that unintended consequences are quickly recognized and minimized. Learning continues as new services and interventions are evaluated and the system continues to respond.

## Data Availability

Not applicable.

## Abbreviations

CDSS: Canadian drugs and substances strategy
DAS: Drug analysis service
HIV: Human immunodeficiency viruses
OAT: Opioid agonist therapy
OPS: Overdose prevention sites
RAAM: Rapid action addiction medicine
SCS: Safer consumption sites
